# Biotherapy in the Adjuvant Treatment of Colorectal Cancer

**DOI:** 10.4021/gr335w

**Published:** 2011-07-20

**Authors:** El Mehdi Tazi, Ismail Essadi, Saber Boutayeb, Hind M’rabti, Hassan Errihani

**Affiliations:** a Department of Medical Oncology, National Institute of Oncology, Rabat, Morocco

**Keywords:** Bevacizumab, Cetuximab, K-ras, FOLFOX, FOLFIRI

## Abstract

The use of adjuvant chemotherapy has improved survival in early-stage colon cancer. Ongoing adjuvant clinical trials are evaluating the addition of targeted therapies to standard chemotherapy regimen. Preliminary results with bevacizumab were disappointing. Also, cetuximab added to chemotherapy does not seem to be better than chemotherapy alone, even in selected wild-type KRAS populations. A better understanding of mechanisms of action of drugs, tumor biology, and predictive biomarkers are needed to design future adjuvant trials.

## Introduction

Colon cancer is the third most common cancer in both men and women, and it is the second leading cause of cancer death in Western countries [1]. Consequently, colon cancer remains a major public health priority. The main prognostic factor for survival or relapse is tumor staging [2]. Surgery is the cornerstone treatment in the case of localized disease (stages I to III). The use of adjuvant therapy is based on the risk of locoregional or distant relapse. This risk is evaluated with 3-year disease-free survival (DFS), which has been recommended by the US Food and Drug Administration Oncology Drugs Advisory Committee as a new regulatory endpoint for full approval in adjuvant colon cancer based on the validation of its surrogacy for 5-year overall survival (OS) [3]. The 3-year DFS in stage III cancer without any postoperative chemotherapy is about 44% - 52% [4, 5].

## Chemotherapy in Colon Cancer

Three cytotoxic drugs are available in the treatment of patients with metastatic colorectal cancer (mCRC), which are fluoropyrimidines, oxaliplatin, and irinotecan. These drugs can be administered either in combination (5-fluorouracil [5-FU]/oxaliplatin or 5-FU/irinotecan) or as monotherapy (fluoropyrimidine alone).

5-Fluorouracil was the first drug to show a survival advantage over surgery alone in adjuvant colon cancer. The 3-year DFS was about 61% - 67% in adjuvant trials using 5-FU [5-10]. This drug was patented in 1957, but only in the early 1990s was it shown that adjuvant chemotherapy with 5-FU and levamisole improved DFS and OS in stage III colon cancer. The Intergroup trial INT-0035 was the first large-scale study to demonstrate a 40% relative reduction in the risk of recurrence and a 33% relative reduction in the overall death rate in patients with stage III colon cancer treated with adjuvant chemotherapy [11]. The International Multicentre Pooled Analysis of Colorectal Cancer Trials compared adjuvant treatment with high-dose 5-FU and leucovorin (LV) with no treatment in nearly 1500 patients, demonstrating a 22% relative risk reduction in mortality in patients with colon cancer [5]. The Mayo Clinic regimen (monthly low-dose LV and bolus 5- FU) significantly improved time to relapse and survival versus observation alone [12]. The Intergroup study INT-0089 demonstrated equivalent efficacy of the modified Roswell Park regimen (weekly high-dose LV and bolus 5-FU) and the Mayo Clinic regimen [13]. Infusional therapy was also tested versus standard intravenous regimens. Biweekly LV and 5-FU bolus plus infusion (LV5FU2), compared with FUFOL (monthly high-dose LV and bolus 5-FU), was investigated in 905 patients with stage II and III colon cancer. Despite the lack of a statistical improvement in DFS [hazard ratio (HR), 1.04; P = .74], LV5FU2 became an accepted standard because of the improved safety profile (P < .001) [14]. The X-ACT (Xeloda in Adjuvant Colon Cancer Therapy) trial randomized 1987 patients with stage III colon cancer to either intravenous monthly LV and bolus 5-FU or oral capecitabine over 6 months. Disease-free survival in the capecitabine arm was at least equivalent to the control arm (HR, 0.87; P < .001) [9]. In the second half of the 1990s, data from several phase III trials in the advanced setting demonstrated that adding irinotecan or oxaliplatin to 5-FU/LV doubled the response rates to around 50% and increased progression-free survival (PFS) and OS in some studies [15-17]. Although modest, these improvements might be of interest to patients with advanced cancer. Thus, both agents have been tested as adjuvant chemotherapy in combination with fluoropyrimidines.

Fluoropyrimidine-and-oxaliplatin combination trials led to a significant advantage in terms of survival in 3 phase III trials [8, 18, 19]. The first was the MOSAIC (Multicenter International Study of Oxaliplatin/5-Fluorouracil/Leucovorin in the Adjuvant Treatment of Colon Cancer) trial, which recruited 2246 patients with stage II and III colon cancer, looking at the addition of oxaliplatin to standard postoperative adjuvant chemotherapy with 5-FU and LV. Adding oxaliplatin resulted in a 23% increase in DFS (HR, 0.77; P = .002). The results were later updated: 5-year DFS rates were 73.3% and 67.4% in the FOLFOX 4 (infusional 5-FU/LV/oxaliplatin) and LV5FU2 groups, respectively (HR, 0.80; P = .003) [20]. The 6-year OS rates were 78.5% and 76.0% in the FOLFOX 4 and LV5FU2 groups, respectively (HR, 0.84; P = .046). Corresponding 6-year OS rates for patients with stage III disease were 72.9% and 68.7%, respectively (HR, 0.80; P = .023). The conclusion of MOSAIC is that adding oxaliplatin to LV5FU2 significantly improved 5-year DFS and 6-year OS in the adjuvant treatment of patients with stage III and high-risk stage II colon cancer and should be considered after surgery. Another oxaliplatin-based regimen, the FLOX regimen, was investigated in the National Surgical Adjuvant Breast and Bowel Project (NSABP) trial C-07, which evaluated the addition of oxaliplatin to weekly bolus 5-FU combined with LV in 2492 patients with stage II and III colon cancer [18]. The extent of benefit in terms of 3-year DFS afforded by oxaliplatin was equivalent to that reported in the MOSAIC study (HR, 0.80; P < .004). With longer follow-up, the DFS advantage in favor of the addition of oxaliplatin remained, and a favorable trend appears to be emerging for OS.

Lastly, the superiority of XELOX (capecitabine/oxaliplatin) as adjuvant treatment over bolus 5-FU/LV has been shown for DFS in 1886 patients with stage III colon cancer in the NO16968 trial, with a 3-year DFS of 71% versus 67% (HR, 0.80; P = .0045) [19]. Unlike in the advanced condition, where the efficacy of oxaliplatin and irinotecan can be considered roughly equivalent [21], 3 studies on the adjuvant use of irinotecan in combination with 5-FU/LV have failed to show superiority over the 5-FU/LV control arm. The Cancer and Leukemia Group B C89803 study compared the IFL (infusional 5-FU/LV/irinotecan) regimen with bolus 5-FU/LV in 1264 patients with stage III colon cancer. Neither DFS (P = .85) nor OS (P = .74) was improved with IFL [22]. The ACCORD-2 study of 400 patients with stage III colon cancer and the PETACC-3/V307 study of 2094 patients with stage III colon cancer used infusional 5-FU regimens as a control arm and the combination of infusional 5-FU and irinotecan as an investigational arm. Neither of these studies met the primary endpoint of superiority of the irinotecan-based chemotherapy over 5-FU alone, with a 3-year DFS of 51% versus 60% (HR, 1.19; P < .22) in the ACCORD2 study [23] and 63% versus 61% (HR, 0.90; P = .106) in the PETACC-3/V307 study [24].

## Targeted Therapies in Colon Cancer

### Bevacizumab

Bevacizumab (Avastin) is a humanized monoclonal antibody (MoAb) targeting vascular endothelial growth factor. Adding bevacizumab to standard chemotherapy (5-FU/irinotecan, 5-FU/oxaliplatin, 5-FU alone) improves outcomes in patients with mCRC [25-27]. Specific bevacizumab-related side effects have been observed: bleeding, hypertension, gastrointestinal (GI) perforation, and arterial thromboembolic events. The addition of bevacizumab has significantly improved the PFS of chemotherapy alone. The magnitude of benefit was higher with irinotecan than with oxaliplatin, and this might be due either to a better synergy or to a more prolonged administration of bevacizumab in the irinotecan trial. Of note, the benefit of bevacizumab appears more pronounced in first-line than in second-line and is not observed in third-line therapy [28, 29]. Angiogenesis plays a role in early-stage colorectal tumor progression [30], justifying the use of angiogenesis inhibitors in the adjuvant setting by preventing angiogenic switch in micrometastases and suppressing vascularization and tumor growth. Vascular endothelial growth factor is the main factor controlling tumor-associated angiogenesis. The NSABP C-08 and the AVANT BO17920 phase III trials have evaluated bevacizumab in combination with an oxaliplatin-based chemotherapy in patients with stage II-III colon cancer. The main differences between these 2 trials are presented in Table 1.

**Table 1:** Differences Between the NASBP C-08 and AVANT Trials

Variable	NASBP C-08	AVANT
Number of arms	2	3
Chemotherapy regimen	mFOLFOX 6	FOLFOX 4, XELOX
Maintenance Bevacizumab	5 mg/Kg every 2 weeks	7.5 mg/Kg every 3 weeks
Analysis	Stage II and III	Stage III

Abbreviations: FOLFOX = infusional 5-fluorouracil/leucovorin/oxaliplatin; NSABP = National Surgical Adjuvant Breast and Bowel Project; XELOX = capecitabine/oxaliplatin.

The NSABP C-08 trial compared the biweekly modified FOLFOX6 regimen (mFOLFOX6; LV 400 mg/m^2^ day 1, oxaliplatin 85 mg/m^2^ day 1, 5-FU bolus 400 mg/m^2^ day 1, 5-FU infusion 2400 mg/m^2^/46 hours) for 6 months with the same regimen with bevacizumab (5 mg/kg every 2 weeks) then bevacizumab alone as maintenance therapy (5 mg/kg every 2 weeks) for an additional 6 months. Contrary to observations in advanced disease, arterial ischemic events, GI perforation, and hemorrhage were not associated with higher frequency in the bevacizumab arm than in the control arm. Toxicities significantly increased with bevacizumab were hypertension, pain, proteinuria, and wound complications [31]. After a follow-up of 36 months, the addition of bevacizumab to mFOLFOX6 did not result in a statistically significant prolongation in DFS, with a 3-year DFS of 77.4% versus 75.5% respectively (HR, 0.87; P = .08), despite a transient benefit in DFS during the first year when bevacizumab was used [32]. No clear rebound effect was observed after 2 years of discontinuation of treatment, with no statistical difference in terms of recurrence, death, or second cancers, which contrasts preclinical data suggesting the possibility that inhibition of angiogenesis could accelerate metastatic behaviour [33-35]. Thus, there are 2 possibilities to explain the C-08 findings (namely, less recurrence during the first year of treatment): either an inhibition of the supposed early angiogenic switch, which was too short to be definitive (if possible), or the prolongation of the PFS in patients who had undetectable micrometastases. If this last hypothesis is true, there is no need to further explore bevacizumab in the adjuvant setting. Because there are not so many new options for adjuvant trials in colon cancer therapy and, furthermore, if the cetuximab adjuvant trials are negative, there will be no advance for the patients for years. If the cetuximab trials are positive, there will be no advance for the patients with mutated KRAS. Thus, there is a need to further study bevacizumab in the adjuvant setting. This should be tested in the NSABP C-12 trial.

The AVANT study B017920 (ClinicalTrials.gov identifier: NCT00112918) compared FOLFOX4 (6 months) versus FOLFOX4 (6 months) with bevacizumab (12 months) or XELOX (6 months) with bevacizumab (12 months) in 3451 patients with stage II or III colon cancer. Primary objectives of this trial were: (1) superiority of bevacizumab plus FOLFOX4 versus FOLFOX4 alone in terms of DFS (patients with stage III disease only), and (2) superiority of bevacizumab plus XELOX versus FOLFOX4 alone in terms of DFS (patients with stage III disease only). The adverse event profile was comparable with the safety profile in metastatic disease and in the NSABP C-08 trial [36]. Pooled safety data of these 2 trials show that hypertension, proteinuria, and wound complications (grade ≥ 3) were significantly increased by the addition of bevacizumab to an oxaliplatin-based chemotherapy in adjuvant setting, whereas neither arterial thrombotic events nor GI perforation or hemorrhage was significantly greater in bevacizumab arms. It should be pointed out that the incidence of venous thrombosis events (grade ≥ 3) was significantly greater with the addition of bevacizumab to chemotherapy (P = .0286) in this pooled analysis, whereas this difference was not statistically significant in the C-08 trial (P = .0635) or in the AVANT study (P = .317).

### Cetuximab

Cetuximab is a chimeric human mouse anti-epidermal growth factor receptor (EGFR) MoAb. It has been studied in combination with oxaliplatin- and irinotecan-based therapy in the palliative setting. It has been shown that only patients with wild-type KRAS tumors respond to cetuximab [37] and can experience a prolongation of PFS. For wild-type KRAS tumors, the addition of cetuximab to either FOLFIRI [CRYSTAL (cetuximab combined with irinotecan in first-line therapy for metastatic colorectal cancer) study] [38] or FOLFOX [OPUS (oxaliplatin and cetuximab in first-line treatment of mCRC) study] [39] showed an improvement in median PFS (9.9 months vs. 8.7 months; P = .02 and 7.7 months vs. 7.2 months; P = .01). However, in the COIN trial, PFS was not prolonged in patients receiving FOLFOX or XELOX plus cetuximab [40]. In this study, KRAS status was prospectively analyzed, which was not the case of the previous studies. In the adjuvant setting, the addition of cetuximab to FOLFOX chemotherapy had no benefit in the US Intergroup N0147 trial (ClinicalTrials.gov: NCT00079274), even in the KRAS population, with a 3-year DFS of 72.3% in the FOLFOX-cetuximab arm versus 75.8% in the FOLFOX arm (HR, 1.2) [41].

Panitumumab is a fully human anti-EGFR MoAb that has also shown a benefit in survival in patients with wild-type KRAS tumors in third-line therapy [42]. More recently, 2 large trials performed in first- and second-line therapy have shown a prolongation of PFS when panitumumab is added to FOLFOX (first-line) or FOLFIRI (second-line) [43, 44].

## Future

### Ongoing trials in the adjuvant setting with bevacizumab

The Eastern Cooperative Oncology Group E5202 trial (ClinicalTrials.gov identifier: NCT00217737; Table 2) is studying FOLFOX with or without bevacizumab in selected patients with stage II disease with microsatellite-stable tumors and loss of heterozygosity. Patients with microsatellite instability and normal 18q receive no treatment after surgery. The aim of this study is to determine prospectively the prognostic value of molecular markers in terms of 3-year DFS (primary endpoint). This study is recruiting patients, with an estimated enrollment of 3610 patients. QUASAR2 (EudraCT identifier: 2005-00029-32; Table 2) is a study comparing 6 months of chemotherapy using capecitabine against capecitabine plus bevacizumab, with the expectation that adding bevacizumab to capecitabine may have the potential for improved relapse-free and overall survival compared with capecitabine alone in patients with stage II and III colon cancer. Recruitment as of January 2010 was 1780 patients, with a target recruitment of 2240 patients.

**Table 2:** Ongoing Phase III Trials in Adjuvant Colon Cancer

Regimen	Stage II	Stage II-III	Stage III
Chemotherapy +/- Bevacizumab	ECOG E5202	NASBP C-08 AVANT QUASAR 2 TOSCA (IDEA)	_
Chemotherapy +/- Cetuximab	_	_	PETACC8 NO147
Chemotherapy +/- Panitumumab	_	BCTU-FOxTROT	_

Abbreviations: BCTU = Birmingham Clinical Trials Unit; ECOG = Eastern Cooperative Oncology Group; FOxTROT = Fluoropyrimidine, Oxaliplatin, and Targeted Receptor Pre-Operative Therapy; NSABP = National Surgical Adjuvant Breast and Bowel Project.

### Ongoing trials in the adjuvant setting with epidermal growth factor receptor inhibitors

The European PETACC8 trials (ClinicalTrials.gov identifier: NCT00265811) are evaluating FOLFOX chemotherapy for 6 months with or without a weekly administration of cetuximab in patients with stage III colon cancer whose tumor was completely removed by surgery (Table 2). The primary endpoint is DFS. The protocol was amended to focus on wild-type KRAS population. The results of this trial are not yet available. The signal used to launch both US NO147 and European PETACC8 phase III trials was given in a first-line phase II study with an overall response rate of 72% [45]. The addition of cetuximab to FOLFOX chemotherapy in early-stage colon cancer had no benefit in the N0147 trial. The results of the PETACC8 trial are not yet available. The Birmingham Clinical Trials Unit FOxTROT (Fluoropyrimidine, Oxaliplatin, and Targeted Receptor Pre-Operative Therapy; ClinicalTrials.gov identifier: NCT00647530) trial is evaluating a neoadjuvant/adjuvant strategy with oxaliplatin-based chemotherapy with or without panitumumab in patients with high-risk colon cancer that can be removed by surgery, with an estimated enrollment of 1050 patients. Primary endpoints are recurrence or persistent disease (including failure of macroscopic disease clearance at primary surgery) rates within the first 2 years and pathologic downstaging as measured by depth of extramural spread among patients allocated to preoperative therapy.

## Conclusion

The goal of an adjuvant therapy is to increase the cure rate in early-stage cancer by eradicating residual micrometastasis. The benefit of adjuvant therapy in colon cancer has been shown with fluoropyrimidines alone, then in combination with oxaliplatin, after having demonstrated an antitumor activity in first-line advanced disease. Despite proven efficacy in metastatic disease, irinotecan in combination with 5-FU could not show any advantage in terms of survival in the adjuvant setting. How do we improve our standard treatment? By adding targeted therapies such as bevacizumab or cetuximab to standard adjuvant chemotherapy? Preliminary results of the first trials failed to demonstrate improvements in survival, even though these drugs were active in metastatic disease. Then, benefit of adjuvant therapy might not be predicted by antitumor activity in the advanced setting. How could we prevent those failures in the adjuvant setting before recruiting a large number of patients? We should certainly improve understanding of mechanisms of action of drugs and tumor biology. Moreover, the optimal schedule for administration of targeted therapies (time, dose, total duration) remains unclear. Looking at biomarkers to select populations or to predict those who can benefit of therapy could be more cost-effective than the too-large adjuvant trials. We should also determine better signal(s) to launch adjuvant trials. Neoadjuvant therapy allowing evaluation of early therapy and biomarkers could provide an answer.

## References

[1] American Cancer Society (2009). Cancer Facts & Figures 2009.

[2] American Joint Committee on Cancer (2009). AJCC Cancer Staging Manual.

[3] Sargent DJ, Wieand HS, Haller DG, Gray R, Benedetti JK, Buyse M, Labianca R (2005). Disease-free survival versus overall survival as a primary end point for adjuvant colon cancer studies: individual patient data from 20,898 patients on 18 randomized trials. J Clin Oncol.

[4] Moertel CG, Fleming TR, Macdonald JS, Haller DG, Laurie JA, Goodman PJ, Ungerleider JS (1990). Levamisole and fluorouracil for adjuvant therapy of resected colon carcinoma. N Engl J Med.

[5] (1995). Efficacy of adjuvant fluorouracil and folinic acid in colon cancer. International Multicentre Pooled Analysis of Colon Cancer Trials (IMPACT) investigators. Lancet.

[6] Fields AL, Keller A, Schwartzberg L, Bernard S, Kardinal C, Cohen A, Schulz J (2009). Adjuvant therapy with the monoclonal antibody Edrecolomab plus fluorouracil-based therapy does not improve overall survival of patients with stage III colon cancer. J Clin Oncol.

[7] Andre T, Colin P, Louvet C, Gamelin E, Bouche O, Achille E, Colbert N (2003). Semimonthly versus monthly regimen of fluorouracil and leucovorin administered for 24 or 36 weeks as adjuvant therapy in stage II and III colon cancer: results of a randomized trial. J Clin Oncol.

[8] Andre T, Boni C, Mounedji-Boudiaf L, Navarro M, Tabernero J, Hickish T, Topham C (2004). Oxaliplatin, fluorouracil, and leucovorin as adjuvant treatment for colon cancer. N Engl J Med.

[9] Twelves C, Wong A, Nowacki MP, Abt M, Burris H, Carrato A, Cassidy J (2005). Capecitabine as adjuvant treatment for stage III colon cancer. N Engl J Med.

[10] Lembersky BC, Wieand HS, Petrelli NJ, O'Connell MJ, Colangelo LH, Smith RE, Seay TE (2006). Oral uracil and tegafur plus leucovorin compared with intravenous fluorouracil and leucovorin in stage II and III carcinoma of the colon: results from National Surgical Adjuvant Breast and Bowel Project Protocol C-06. J Clin Oncol.

[11] Moertel CG, Fleming TR, Macdonald JS, Haller DG, Laurie JA, Tangen CM, Ungerleider JS (1995). Intergroup study of fluorouracil plus levamisole as adjuvant therapy for stage II/Dukes' B2 colon cancer. J Clin Oncol.

[12] O'Connell MJ, Mailliard JA, Kahn MJ, Macdonald JS, Haller DG, Mayer RJ, Wieand HS (1997). Controlled trial of fluorouracil and low-dose leucovorin given for 6 months as postoperative adjuvant therapy for colon cancer. J Clin Oncol.

[13] Haller DG, Catalano PJ, Macdonald JS, O'Rourke MA, Frontiera MS, Jackson DV, Mayer RJ (2005). Phase III study of fluorouracil, leucovorin, and levamisole in high-risk stage II and III colon cancer: final report of Intergroup 0089. J Clin Oncol.

[14] de Gramont A, Bosset JF, Milan C, Rougier P, Bouche O, Etienne PL, Morvan F (1997). Randomized trial comparing monthly low-dose leucovorin and fluorouracil bolus with bimonthly high-dose leucovorin and fluorouracil bolus plus continuous infusion for advanced colorectal cancer: a French intergroup study. J Clin Oncol.

[15] de Gramont A, Figer A, Seymour M, Homerin M, Hmissi A, Cassidy J, Boni C (2000). Leucovorin and fluorouracil with or without oxaliplatin as first-line treatment in advanced colorectal cancer. J Clin Oncol.

[16] Saltz LB, Cox JV, Blanke C, Rosen LS, Fehrenbacher L, Moore MJ, Maroun JA (2000). Irinotecan plus fluorouracil and leucovorin for metastatic colorectal cancer. Irinotecan Study Group. N Engl J Med.

[17] Douillard JY, Cunningham D, Roth AD, Navarro M, James RD, Karasek P, Jandik P (2000). Irinotecan combined with fluorouracil compared with fluorouracil alone as first-line treatment for metastatic colorectal cancer: a multicentre randomised trial. Lancet.

[18] Kuebler JP, Wieand HS, O'Connell MJ, Smith RE, Colangelo LH, Yothers G, Petrelli NJ (2007). Oxaliplatin combined with weekly bolus fluorouracil and leucovorin as surgical adjuvant chemotherapy for stage II and III colon cancer: results from NSABP C-07. J Clin Oncol.

[19] Haller DG, Tabernero J, Maroun J (2009). First efficacy findings from a randomized phase III trial of capecitabine + oxaliplatin vs. bolus 5-FU/LV for stage III colon cancer (NO16968/XELOXA study). Eur J Cancer Suppl.

[20] Andre T, Boni C, Navarro M, Tabernero J, Hickish T, Topham C, Bonetti A (2009). Improved overall survival with oxaliplatin, fluorouracil, and leucovorin as adjuvant treatment in stage II or III colon cancer in the MOSAIC trial. J Clin Oncol.

[21] Tournigand C, Andre T, Achille E, Lledo G, Flesh M, Mery-Mignard D, Quinaux E (2004). FOLFIRI followed by FOLFOX6 or the reverse sequence in advanced colorectal cancer: a randomized GERCOR study. J Clin Oncol.

[22] Saltz LB, Niedzwiecki D, Hollis D, Goldberg RM, Hantel A, Thomas JP, Fields AL (2007). Irinotecan fluorouracil plus leucovorin is not superior to fluorouracil plus leucovorin alone as adjuvant treatment for stage III colon cancer: results of CALGB 89803. J Clin Oncol.

[23] Ychou M, Hohenberger W, Thezenas S, Navarro M, Maurel J, Bokemeyer C, Shacham-Shmueli E (2009). A randomized phase III study comparing adjuvant 5-fluorouracil/folinic acid with FOLFIRI in patients following complete resection of liver metastases from colorectal cancer. Ann Oncol.

[24] Van Cutsem E, Labianca R, Bodoky G, Barone C, Aranda E, Nordlinger B, Topham C (2009). Randomized phase III trial comparing biweekly infusional fluorouracil/leucovorin alone or with irinotecan in the adjuvant treatment of stage III colon cancer: PETACC-3. J Clin Oncol.

[25] Saltz LB, Clarke S, Diaz-Rubio E, Scheithauer W, Figer A, Wong R, Koski S (2008). Bevacizumab in combination with oxaliplatin-based chemotherapy as first-line therapy in metastatic colorectal cancer: a randomized phase III study. J Clin Oncol.

[26] Hurwitz H, Fehrenbacher L, Novotny W, Cartwright T, Hainsworth J, Heim W, Berlin J (2004). Bevacizumab plus irinotecan, fluorouracil, and leucovorin for metastatic colorectal cancer. N Engl J Med.

[27] Kabbinavar FF, Schulz J, McCleod M, Patel T, Hamm JT, Hecht JR, Mass R (2005). Addition of bevacizumab to bolus fluorouracil and leucovorin in first-line metastatic colorectal cancer: results of a randomized phase II trial. J Clin Oncol.

[28] Giantonio BJ, Catalano PJ, Meropol NJ, O'Dwyer PJ, Mitchell EP, Alberts SR, Schwartz MA (2007). Bevacizumab in combination with oxaliplatin, fluorouracil, and leucovorin (FOLFOX4) for previously treated metastatic colorectal cancer: results from the Eastern Cooperative Oncology Group Study E3200. J Clin Oncol.

[29] Chen HX, Mooney M, Boron M, Vena D, Mosby K, Grochow L, Jaffe C (2006). Phase II multicenter trial of bevacizumab plus fluorouracil and leucovorin in patients with advanced refractory colorectal cancer: an NCI Treatment Referral Center Trial TRC-0301. J Clin Oncol.

[30] Poon RT, Fan ST, Wong J (2001). Clinical implications of circulating angiogenic factors in cancer patients. J Clin Oncol.

[31] Allegra CJ, Yothers G, O'Connell MJ, Sharif S, Colangelo LH, Lopa SH, Petrelli NJ (2009). Initial safety report of NSABP C-08: A randomized phase III study of modified FOLFOX6 with or without bevacizumab for the adjuvant treatment of patients with stage II or III colon cancer. J Clin Oncol.

[32] Wolmark N, Yothers G, O’Connell MJ (2009). A phase III trial comparing mFOLFOX6 to mFOLFOX6 plus bevacizumab in stage II or III carcinoma of the colon: results of NSABP Protocol C-08. J Clin Oncol.

[33] Loges S, Mazzone M, Hohensinner P, Carmeliet P (2009). Silencing or fueling metastasis with VEGF inhibitors: antiangiogenesis revisited. Cancer Cell.

[34] Ebos JM, Lee CR, Cruz-Munoz W, Bjarnason GA, Christensen JG, Kerbel RS (2009). Accelerated metastasis after short-term treatment with a potent inhibitor of tumor angiogenesis. Cancer Cell.

[35] Paez-Ribes M, Allen E, Hudock J, Takeda T, Okuyama H, Vinals F, Inoue M (2009). Antiangiogenic therapy elicits malignant progression of tumors to increased local invasion and distant metastasis. Cancer Cell.

[36] Hoff P, Clarke S, Cunningham D (2009). A three-arm phase III randomized trial of FOLFOX-4 vs. FOLFOX-4 plus bevacizumab vs. XELOX plus bevacizumab in the adjuvant treatment of patients with stage III or high-risk stage II colon cancer: results of the interim safety analysis of the AVANT trial. Eur J Cancer Suppl.

[37] Lievre A, Bachet JB, Boige V, Cayre A, Le Corre D, Buc E, Ychou M (2008). KRAS mutations as an independent prognostic factor in patients with advanced colorectal cancer treated with cetuximab. J Clin Oncol.

[38] Van Cutsem E, Kohne CH, Hitre E, Zaluski J, Chang Chien CR, Makhson A, D'Haens G (2009). Cetuximab and chemotherapy as initial treatment for metastatic colorectal cancer. N Engl J Med.

[39] Bokemeyer C, Bondarenko I, Makhson A, Hartmann JT, Aparicio J, de Braud F, Donea S (2009). Fluorouracil, leucovorin, and oxaliplatin with and without cetuximab in the first-line treatment of metastatic colorectal cancer. J Clin Oncol.

[40] Maughan T, Adams RA, Smith CG (2009). Addition of cetuximab to oxaliplatin-based combination chemotherapy (CT) in patients with KRAS wild-type advanced colorectal cancer (ACRC): a randomised superiority trial (MRC COIN). Eur J Cancer Suppl.

[41] Alberts SR, Sargent DJ, Smyrk TC (2010). Adjuvant mFOLFOX6 with or without cetuxiumab (Cmab) in KRAS wild-type (WT) patients (pts) with resected stage III colon cancer (CC): Results from NCCTG intergroup phase III trial N0147. J Clin Oncol.

[42] Van Cutsem E, Peeters M, Siena S, Humblet Y, Hendlisz A, Neyns B, Canon JL (2007). Open-label phase III trial of panitumumab plus best supportive care compared with best supportive care alone in patients with chemotherapy-refractory metastatic colorectal cancer. J Clin Oncol.

[43] Douillard JY, Siena S, Cassidy J (2009). Randomized phase 3 study of panitumumab with FOLFOX4 vs FOLFOX4 alone as first-line treatment in patients with metastatic colorectal cancer: the PRIME trial. Eur J Cancer Suppl.

[44] Peeters M, Price T, Hotko Y (2009). Randomized phase III study of panitumumab with FOLFIRI vs FOLFIRI alone as second-line treatment in patients with metastatic colorectal cancer. Eur J Cancer Suppl.

[45] Tabernero J, Van Cutsem E, Diaz-Rubio E, Cervantes A, Humblet Y, Andre T, Van Laethem JL (2007). Phase II trial of cetuximab in combination with fluorouracil, leucovorin, and oxaliplatin in the first-line treatment of metastatic colorectal cancer. J Clin Oncol.

